# Supplementary material for: Splice-Junction-Based Mapping of Alternative Isoforms in the Human Proteome
Source: Cell Rep. Author manuscript; Available in PMC 2020 Jan 15. (PMC6961840; doi:10.1016/j.celrep.2019.11.026)
Supplement: 2 [file NIHMS1546469-supplement-2.zip › DF1/PXD010154/Liver/Liver_60_LRCH4_SPFPAAGGEEAAATTSVPGSPGLPGR.pdf]

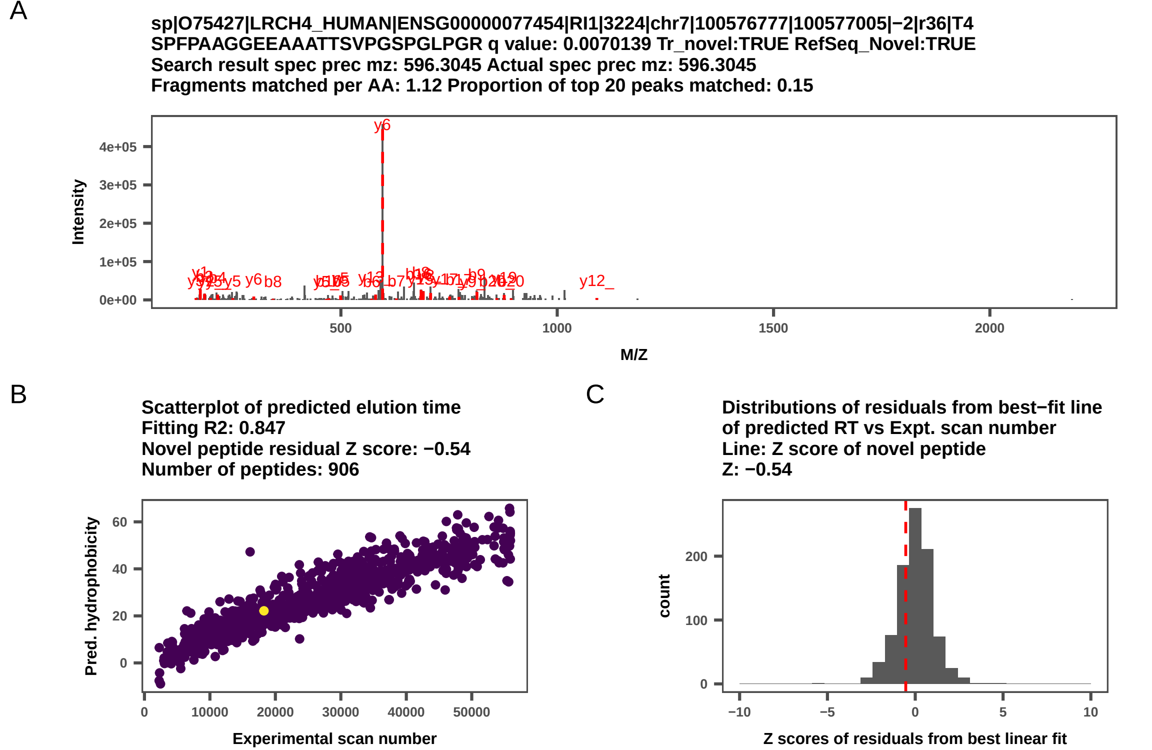
A
sp|O75427|LRCH4_HUMAN|ENSG00000077454|RI1|3224|chr7|100576777|100577005|−2|r36|T4
SPFPAAGGEEAAATTSVPGSPGLPGR q value: 0.0070139 Tr_novel:TRUE RefSeq_Novel:TRUE
Search result spec prec mz: 596.3045 Actual spec prec mz: 596.3045
Fragments matched per AA: 1.12 Proportion of top 20 peaks matched: 0.15
y6
y12_
Intensity
4e+05
3e+05
2e+05
1e+05
0e+00
500
1000
1500
2000
M/Z
B
Scatterplot of predicted elution time
Fitting R2: 0.847
Novel peptide residual Z score: −0.54
Number of peptides: 906
Pred. hydrophobicity
60
40
20
0
0
10000
20000
30000
40000
50000
Experimental scan number
C
Distributions of residuals from best−fit line
of predicted RT vs Expt. scan number
Line: Z score of novel peptide
Z: −0.54
count
200
100
0
−10
−5
0
5
10
Z scores of residuals from best linear fit
